# Lack of Bmal1 leads to changes in rhythmicity and impairs motivation towards natural stimuli

**DOI:** 10.1098/rsob.240051

**Published:** 2024-07-24

**Authors:** Paula Berbegal-Sáez, Ines Gallego-Landin, Javier Macía, Laia Alegre-Zurano, Adriana Castro-Zavala, Patrick-Simon Welz, Salvador A. Benitah, Olga Valverde

**Affiliations:** ^1^ Department of Medicine and Life Sciences (MELIS), Neurobiology of Behaviour Research Group (GReNeC-NeuroBio), Universitat Pompeu Fabra, Barcelona, Spain; ^2^ Department of Medicine and Life Sciences (MELIS), Synthetic Biology for Biomedical Applications, Universitat Pompeu Fabra, Barcelona, Spain; ^3^ Program in Cancer Research, Hospital del Mar Research Institute (IMIM), Barcelona, Spain; ^4^ Institute for Research in Biomedicine (IRB Barcelona), Barcelona Institute of Science and Technology, Barcelon 08028, Spain; ^5^ Catalan Institution for Research and Advanced Studies (ICREA), Barcelona, Spain; ^6^ Neuroscience Research Program, Hospital del Mar Research Institute (IMIM), Barcelona, Spain

**Keywords:** reward system, Bmal1, daily rhythms, circadian disruption, natural reinforcer

## Abstract

Maintaining proper circadian rhythms is essential for coordinating biological functions in mammals. This study investigates the effects of daily arrhythmicity using Bmal1-knockout (KO) mice as a model, aiming to understand behavioural and motivational implications. By employing a new mathematical analysis based on entropy divergence, we identified disrupted intricate activity patterns in mice derived by the complete absence of BMAL1 and quantified the difference regarding the activity oscillation’s complexity. Changes in locomotor activity coincided with disturbances in circadian gene expression patterns. Additionally, we found a dysregulated gene expression profile particularly in brain nuclei like the ventral striatum, impacting genes related to reward and motivation. Further investigation revealed that arrhythmic mice exhibited heightened motivation for food and water rewards, indicating a link between circadian disruptions and the reward system. This research sheds light on how circadian clock alterations impact the gene expression regulating the reward system and how this, in turn, can lead to altered seeking behaviour and motivation for natural rewards. In summary, the present study contributes to our understanding of how reward processing is under the regulation of circadian clock machinery.

## 1. Introduction

The circadian clock controls the body’s natural rhythms by synchronizing them to the environment over 24 h. Essential for maintaining internal temporal order, these molecular rhythms play a critical role in regulating a variety of biological processes, including sleep-wake cycles, hormone production and secretion, metabolic activities and behaviour [1–3]. The core architecture of the mammalian circadian clock relies on a complex molecular network featuring key components such as the brain and muscle ARNT-like 1 (BMAL1) protein produced by the *Arntl* gene, and circadian locomotor output cycles kaput (*Clock*), or the paralog neuronal PAS domain protein 2 (*Npas2*). These proteins heterodimerize, forming the CLOCK:BMAL1 or, alternatively, the NPAS2:BMAL1 complexes. These act as transcription factors binding to the E-box regions upstream, thus promoting the transcription of the gene families Period and Cryptochrome (*Per* and *Cry*). These proteins will also heterodimerize into a PER:CRY complex that will inhibit the activity of the CLOCK:BMAL1 dimer, thus abolishing their own expression and establishing a negative transcriptional loop. Additional genes, such as *Rev-erb* and *Rora*, form a secondary self-regulation loop by repressing or enhancing *Bmal1* expression, respectively, which is known as the BMAL1 loop [4,5]. Moreover, the CLOCK:BMAL1 transcriptional factor binds to multiple E-boxes, controlling the transcription of numerous clock-controlled genes (CCGs) [6]. Therefore, regulating *Bmal1* expression is crucial for maintaining normal circadian rhythms in mammals. In this regard, the *Bmal1* knockout (Bmal1-KO) mouse line has emerged as an optimal animal model to study how arrhythmia occurs in the circadian clock and how it can affect different systems [7].

Notably, disruption of rhythmicity has also been linked to changes in motivation and reward modulation. At a molecular level, studies in both humans and mice have linked genetic modifications in *Bmal1*, *Per* and *Clock* genes to increased drug use and altered reward processing [8–12]. Alternatively, disruption of circadian rhythms can also be induced by environmental factors, many of which have long been described and researched in humans. For example, sleep deprivation, shift work or jetlag are associated to pathological states that impact reward and motivational characteristics such as food and drug intake [13,14].

In the realm of motivated behaviour, the dopaminergic system plays a central role within the mesocorticolimbic circuit. This pivotal pathway extends from the ventral tegmental area (VTA) to the ventral striatum (vSTR), including the nucleus accumbens (NAc) and projections to the prefrontal cortex (PFC). Interestingly, dopamine (DA) production and degradation are primarily regulated by the circadian cycle through the clock-controlled catabolic enzymes monoamino oxidase A enzyme (MAOA) and monoamino oxidase B enzyme (MAOB), indicating a close interplay between these systems [15,16]. Furthermore, the dopaminergic system is subject to regulation by different neuromodulators, such as the endocannabinoid system, particularly through cannabinoid receptor 1 (CB1)-mediated actions [17].

The reward system responds to different types of cues in comparable and different ways [18]. Food serves as a primary natural reward that acts as an essential stimulus for motivated responses and is also crucial for maintaining a proper metabolic state. Here, the hypothalamus (HT), an integrated energy-sensing centre, manages caloric and nutritional needs by sensing macronutrients and circulating regulatory hormones, neuropeptides and neuromodulators like leptin, ghrelin, orexin/hypocretin, insulin, neuropeptide Y (NPY) and endocannabinoids [19,20]. Besides, this brain region contains the suprachiasmatic nucleus (SCN), the so-called central pacemaker [4]. The SCN serves as a critical component that synchronizes metabolic regulation with daily changes in the environment by integrating information from circadian cues such as light or food intake. This integration allows for the coordination of appropriate behaviours in response to environmental stimuli. Some studies have evaluated whether a proper synchronization of the feeding pattern could potentially prevent reward impairments [21,22]. Together, the regulation of feeding behaviour and energy homeostasis is a complex process that requires the integration of a timekeeping system and feeding signals from the HT, but also motivational feeding signals from the mesolimbic pathways. Furthermore, the integration of homeostatic and reward-related feeding signals is facilitated by other higher-level decision-making centres, including the medial prefrontal cortex (mPFC), hippocampus and amygdala, which are linked to the neurocircuitry of the motivation control [23, 24].

While prior research has predominantly focused on investigating circadian disruption due to the lack of *Bmal1* gene in the context of drugs of abuse such as cocaine [8] or alcohol consumption [10,25], this study aims to elucidate the impact of physiological arrhythmicity on homeostatic motivated behaviour. To achieve this, we have utilized Bmal1-KO mice as an appropriate model for circadian arrhythmicity. Locomotor activity, a key circadian parameter, was analysed using a novel mathematical method. In addition, we employed Kronos software [26] to conduct a classical analysis and assess rhythmicity in biological data. Additionally, we delved into the brain gene expression profile of these mice, focusing on the systems influencing the rewarding value of feeding behaviour. Subsequently, we examined the motivational behaviour of the animals through self-administration (SA) paradigms, using food and water as natural rewards. Our comprehensive approach unveiled the complex patterns that characterize circadian disruption consequences in behaviour within our mouse model.

## 2. Material and methods

### 2.1. Experimental model and study participant details

#### 2.1.1. Mice

Mice were obtained from the heterozygous C57BL/6 (Bmal1(+/−)) breeding colonies, kindly donated by Stem Cells and Cancer Lab at the IRB (Barcelona) and housed at UBIOMEX-PRBB. Animal rooms were maintained in a 12 h light/dark cycle (lights on at 7.30 am), with a constant temperature (21 ± 1°C) and humidity (55% ± 10%). After weaning, mice were grouped, housed and genotyped as homozygous Bmal1-KO (Bmal1(−/−)), heterozygous (Bmal1 (+/−)) or wild-type (WT) (Bmal1 (+/+)). Only Bmal1-KO and WT animals at postnatal day 70 were used in the experiments. Both sexes, males and females, were used equally.

### 2.2. Method details

#### 2.2.1. Experimental design

We used different batches of animals as indicated for each experimental procedure. Both male and female mice were analysed as a single group throughout all experiments since no significant differences between sexes were observed. First, we analysed the daily rhythmicity of the animals through continuous and spontaneous locomotor activity measurements for seven consecutive days (*n* = 11 per group) under a light–dark cycle. Time-series data were analysed both with a new mathematical analysis based on the variation of accumulated entropy (VAE) and Kronos software.

A second group of Bmal1-KO and WT animals was used for the analysis of the rhythmicity of main clock genes *Per2*, *Cry2*, *Clock* and *Rev-erba* through a time-course gene expression analysis. Mice under ad libitum conditions (basal conditions) (*n* = 3–4 per time-point and experimental group) were sacrificed every 4 h over a 24 h period (Zeitgeber time [ZT] 2, 6, 10, 14, 18 and 22, considering that ZT0 and ZT12 represent the onset of the light and dark phases, respectively) during which, samples from HT were collected for quantitative PCR (qPCR) experiments. Circadian rhythmicity of gene expression data were analysed using Kronos, a computational tool to assess biological rhythms.

For the evaluation of differential gene expression between the WT and Bmal1-KO, a custom OpenArray platform was used. Animals were sacrificed (*n* = 6 per group) at the same time-point (ZT18), and tissue corresponding to HT, mPFC and vSTR was collected. Then, the mRNA was extracted, followed by RT-PCR and the resulting cDNA was analysed in a customized OpenArray plate.

In order to evaluate the motivational behaviour of the animals, we exposed them to operant behaviour paradigms where seeking and motivation for reinforcement were assessed. We conducted two different SA experiments using different animal batches. In the first SA (WT, *n* = 22; Bmal1-KO, *n* = 14), we used food as a reinforcer, while in the second one (WT, *n* = 14; Bmal1-KO, *n* = 14), water was used as a non-caloric reinforcer [27] to determine if the increase in reinforcement seeking could be affected by changes in metabolic balance resulting from the caloric component of the food. All the animals followed similar protocols with minor modifications, and only food SA animals underwent the demanding task as an extra behavioural evaluation test. All the SA sessions were performed throughout the dark phase of the light-dark cycle.

After the SA procedures, mice were sacrificed and tissue from HT and vSTR were dissected at ZT18 for molecular gene expression analysis with qPCR (WT, *n* = 15; Bmal1-KO, *n* = 9). We examined genes related to circadian rhythms and metabolic control within the HT (*AgRP, Npy, Avp, Vip* and *Hcrtr1*) and reward-related control genes within the vSTR (*Drd1, Drd2, Maoa, Maob* and *Cnr1*).

#### 2.2.2. Spontaneous locomotor activity recording

For the assessment of spontaneous locomotion throughout the light/dark cycle (L:12, D:12), each mouse was individually housed in a cage (32 × 17 × 14 cm). Over seven consecutive days, the locomotor activity of each animal was recorded using the Panlab infrared (IR) Actimeter (LE881 IR, Panlab s.l.u., Barcelona, Spain) and the SEDACOM software. The motor activity (MA) of a mouse was quantified as the number of counts per 15-minure intervals for VAE analyses and the accumulated counts per hour for Kronos evaluation (MA h^−1^).

#### 2.2.3. Food self-administration procedure

Mice underwent food deprivation conditions for two days before the beginning of the SA procedure, maintaining 95% of their initial weight as previously reported [28]. Throughout the evaluation of food operant behaviour, animals remained under the same food deprivation regime and were weighed after each session (see electronic supplementary material, figure S1).

Mice were trained in the operant chambers (17.8 × 15.2 × 18.4 cm) (Med Associates, St Albans, VT, USA) to nose-poke for standard non-flavoured food pellets (Noyes Precision Pellets, Research Diets Inc., USA) during 2-h sessions. Nose-poking in the active hole of the chamber was rewarded with one food pellet and paired with a light cue. Inactive nose-pokes yielded no output. For the first 10 days of the procedure, mice were trained under a fixed ratio (FR)1 schedule of reinforcement, where a nose-poke in the active hole resulted in the delivery of a single pellet. This was followed by 5 days under an FR3 schedule. Subsequently, a progressive ratio (PR) test was conducted, wherein the response requirement to earn a single reward escalated according to the following series: 1−2−3−5−12−18−27−40−60−90−135−200−300−450−675−1000. The breakpoint was defined as the number of nose-pokes required to obtain the last pellet in the PR schedule. Finally, mice completed a demand task test modified from previous studies [29] where the criteria for earning a pellet followed an ascending trend every 10 min: 1, 2, 4, 6, 8, 11, 14, 18, 21, 28, 35 and 42.

#### 2.2.4. Oral water self-administration procedure

Modified from [28], 2 days before beginning the experiment, access to water was restricted to 1 h per day (in the dark phase of the cycle). This limitation aimed to increase the motivation for nose-poking. It was maintained throughout the SA procedure, during which mice had access for one hour again after the SA session. This was implemented to prevent dehydration in the animals, regardless of their performance in the procedure. Again, the animal’s body weight was monitored daily after each session (see electronic supplementary material). Also, food was available ad libitum before and along the water SA procedure and throughout the day. Mice were trained to receive water reinforcement under a fixed ratio (FR)1 (10 days) and FR3 (5 days) schedules of reinforcement. Twenty-four hours later, they underwent the PR test, mirroring the protocol used for food SA. The liquid dipper delivered water in 23  μl over 20 s, which was considered as a time-out period in which active nose-pokes had no consequences. Each session finished either after 180 reinforcers were delivered or after 1 h had passed.

#### 2.2.5. Tissue collection and RNA expression analyses

Mice were sacrificed by cervical dislocation followed by immediate brain extraction. Brain samples were collected using a 1 mm brain matrix pre-cooled at 4°C, then placed in dry iced and immediately stored at −80°C. Total mRNA was extracted from brain tissue using TRIzol reagent, following the manufacturer’s instructions and isopropanol for RNA precipitation. Then, cDNA was synthesized by reverse transcription of total mRNA. Time point gene expression and post-behavioural molecular analysis were assessed through quantitative qPCR. The cDNA template was mixed with the primers (see electronic supplementary material, table S1) and SYBR Green PCR Master Mix. The expression level of each gene of interest within each sample was normalized against glyceraldehyde 3‐phosphate dehydrogenase (*Gapdh*) and expressed relative to either WT group or to ZT2 of

WT group for time-point gene expression. The fold change in gene expression of Bmal1-KO animals compared to controls was calculated using the 2^−ΔΔCt^ method.

#### 2.2.6. OpenArray technology

To perform the OpenArray analyses, 2.5 μl of cDNA sample was combined with 2.5 μl TaqMan OpenArray Real-Time Master Mix (Thermo Fisher no. 4462159) and loaded into a single well of a 384-well plate [30]. Custom OpenArray plates were then automatically loaded using the AccuFill System (AccuFill System User Guide, PN4456986) and run in QuantStudio 12K. Amplification of the sequence of interest was normalized to reference endogenous genes, specifically, the geometric mean of *Actb*, *Gapdh* and *Hprt1*. Fold-change values were calculated using the ΔΔCt method. Data were analysed with the ThermoFisher Connect cloud web tool and Microsoft Excel.

### 2.3. Quantification and statistical analysis

#### 2.3.1. Entropy divergence analyses

For this analysis, we introduce a new analysis method called VAE. This method allows for determining the differences, in terms of information complexity, between two-time series. Briefly (see electronic supplementary material for extended details), first, the discrete Fourier transform is applied to the different time series to obtain their *n* basic frequency components (i.e. harmonics), with their corresponding phases and amplitudes. Each of these harmonics contains a part of the total information contained in the original time series. Subsequently, a subset of the first *M* harmonics (*n* ≥ *M* ≥ 0) is selected and the inverse discrete Fourier transform is applied to this subset to obtain a partial reconstruction of the original time series. For each of these subsets of *M* harmonics, the Shannon information entropy [ 31] *E*_*M*_ is calculated. In summary, a partial reconstruction of a time series using the first *M* harmonics of its decomposition in the Fourier spectrum will contain an informational complexity determined by *E*_*M*_. As the size of the subset of *M* harmonics increases, the reconstructed time series will be closer to the original and the contained information, determined by *E*_*M*_, will also be greater, i.e. the values of *E*_*M*_ will increase as the size of the subset *M* used to reconstruct the time series increases. For the time series obtained experimentally with the Bmal1-KO animals and WT, we define the VAE for a given subset of harmonics *M* as


(2.1) $$VAE\left(M\right)={E_{M}^{Bmal1-KO}-E}_{M}^{WT}$$

with $E_{M}^{WT}$ being the entropy associated with the subset formed by the first *M* harmonics of the time series obtained with the control animals, and $E_{M}^{Bmal1-KO}$ corresponding to that obtained with the Bmal1-KO mice. If beyond a certain critical value M^*^, the values of VAE stabilize, this indicates that considering more harmonics in the reconstruction of the time series, i.e. beyond a certain critical value (M*), the contributions to the accumulated entropy would not add significant information of the phenotypic characteristics of the mice that influence the obtained data series. This subset of M^*^ harmonics contains the essential information that characterizes the circadian cycle in the function of the specific characteristics of the animals. Consequently, applying the inverse discrete Fourier transform to this subset is sufficient to reconstruct the time series of the circadian cycle determined by the specific phenotypic characteristics of the animals and discard other contributions to the time series, obtained experimentally, that depend on other factors. Furthermore, the value of VAE(M^*^) can be considered a metric that allows quantifying the distance, in terms of informational complexity contained in their circadian cycles, between the two sets of animals, WT and Bmal1-KO.

#### 2.3.2. Kronos

We harnessed Kronos, a computational tool by Bastiaanssen *et al.* [26], to analyse biological rhythmicity efficiently in our study. This software specializes in evaluating circadian rhythms within biological datasets. Kronos proved instrumental in our study, streamlining rhythmicity analysis and providing nuanced insights into oscillatory responses. Kronos predicts sinusoid curves for each variable based on a specified period, generating a kronosOut object with valuable outputs: variance proportion explained by predicted curves, corresponding *p*-values, acrophase and amplitude. It also offers model details beneficial for prediction and statistical applications.

To compare rhythmicity between groups, Kronos employs a generalized linear model on user-defined categorical predictors, decomposed sine and cosine components and interactions. The resulting kronosOut object encompasses interaction details, aggregating factor-related *p*-values with Bonferroni’s adjustment for the lowest adjusted *p*‐value. Additionally, it facilitates pairwise comparisons, distinguishing overall differences from group-specific rhythmicity variations. Adhering to Kronos' original methods, we estimated rhythm characteristics, focusing on vital 24-h cycles crucial for maintaining health and synchronizing physiological processes. For locomotor activity data, we employed the accumulated counts per hour and calculated the mean of each recorded day for each individual animal.

#### 2.3.3. Statistical analysis

Normality (D'Agostino–Pearson and Kolmogorov–Smirnov tests), heteroscedasticity and homoscedasticity were assessed for all data sets. For single-factor, two-group analyses involving parametric variables, we employed unpaired student’s *t-*tests. The study always considers the sex variable. If there are no differences, both sexes are analysed as a single population. When an experimental condition followed a within-subject design a two-way ANOVA with repeated measures was conducted.

Demand curves were analysed using the exponential model [32]: logQ = logQ_0_ + k (e − αQ_0_C − 1). In the exponential demand curve graphs, food pellet demand is plotted as a function of the price. Q_0_ represents consumption at a minimum price, while α measures behavioural elasticity, assessing demand responsiveness to commodity price shifts [33]. For the analysis of P_max_, we used the Excel calculator provided by Kaplan *et al*. [34]. P_max_, known as the point of unit elasticity, is a metric used to evaluate motivation. The extra sum-of-squares F-test was used to evaluate the fitness of the curves. This process was used to determine whether a single curve is sufficient to fit data from both WT and Bmal1-KO mice or whether distinct curves are more appropriate for the two datasets, thereby indicating a distinct behaviour between groups.

## 3. Results

### 3.1. BMAL1 deficient mice show daily arrhythmia in spontaneous locomotor activity and gene oscillation

**Figure 1.**
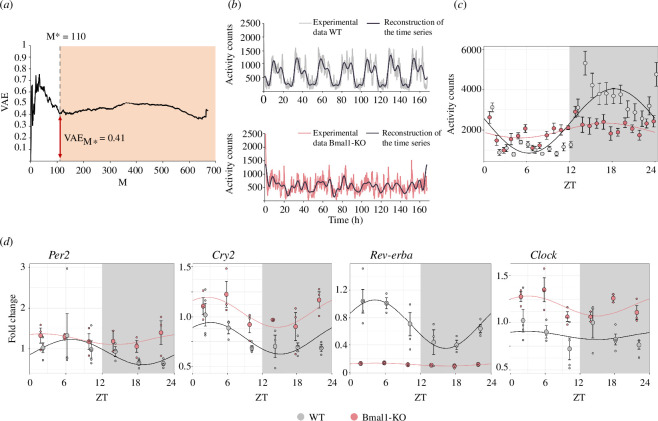
Assessment of daily arrhythmicity of Bmal1-KO mice. (*a*). VAE values as a function of the number of Fourier components M used in their calculation. (*b*) Reconstruction of the time series of locomotor activity in wild-type and Bmal1-KO mice using only the first M* = 110 Fourier components (*n* = 11 mice per group). (*c*) Kronos analyses of locomotor activity data of WT and Bmal1-KO mice. Activity counts were collapsed to 1 h and mean activity along the seven recorded days was calculated for each individual animal. (*d*). Graphical representation of time-course gene expression data of *Per2*, *Cry2*, *Rev-erba* and *Clock* genes within the hypothalamus (*n* = 3 mice per group and time point). Graphs resulting from Kronos software show the cosine-fitted curves and SD from WT or Bmal1-KO mice. Period = 24 h, 12 h light (white zone), 12 h darkness (grey zone). ZT, Zeitgeber time, where ZT0 lights turn on. VAE, variation of accumulated entropy.

To quantify daily arrhythmia status in locomotion or how one group differed from another by this parameter, we pioneered the use of VAE based on the assumption that information in the corresponding time series of mice’s locomotor activity is altered as a result of disruptions to internal rhythms. From the decomposition and mathematical processing of experimental data (see §2) VAE(*M*) is calculated. The results obtained show two distinct regions of behaviour (figure 1*a*). In the first region (white area), VAE values changed significantly as the number of components (*M*) increased. This can be interpreted as each component of the subset *M* provides different information depending on whether it corresponds to the wild-type group or the Bmal1-KO animals. Therefore, this difference in the contained information is dependent on the specific characteristics of the analysed experimental group. In the second region (coloured area), starting from the value M* = 110, it is observed that the VAE value exhibits significantly smaller variations, indicating that the new Fourier components added to reconstruct the curve of experimental data provide a similar amount of information to both time series. Therefore, they do not strongly rely on the attributes of each analysed group and may relate to other shared factors. As a result, they do not significantly offer insights into daily rhythm variation. Figure 1*b* displays the original time series experimentally measured, S^WT^ (t) and S^Bmal1-KO^ (t). In these figures, the blue lines represent the reconstruction of the time series, applying the inverse Fourier transform, considering only the first M* Fourier components, which contain information dependent on the characteristics of each experimental group. As observed, the Bmal1-KO also displayed reduced locomotor activity. Moreover, we obtained the value of VAE M* = 0.41, a measure of the difference in the information complexity contained in the time series of locomotor activity. This positive value indicates that Bmal1-KO mice exhibit a highly complex circadian dynamic regarding the amount of information contained in the time series compared to WT mice. This numeric value quantifies the disorganization and randomness in the pattern that describes both the experimental and the reconstruction data.

The analysis of the locomotor activity data using Kronos software (figure 1*c*) revealed a reduction in locomotor activity in Bmal1-KO mice given by the amplitude of the curve (WT: 1594.1; Bmal1-KO: 368.4), which is consistent with the observations provided by the VAE analysis. Interestingly, both groups showed similar acrophases (WT: 18.3; Bmal1-KO: 17.1) and demonstrated circadian oscillations in their activity patterns (WT: *p* = 5.39 × 10^−27^; Bmal1-KO: *p* = 2.07 × 10^−5^). However, pairwise comparisons revealed statistically significant differences in oscillation patterns between the two groups (*p* = 6.45 × 10^−16^). This suggests that KO mice still exhibit a dim rhythmic activity pattern under light–dark conditions.

Furthermore, the analysis of gene oscillation using the Kronos software facilitated a detailed analysis of rhythmicity within our experimental framework (see electronic supplementary material for the statistical details), leading to distinct observations regarding gene oscillations in WT and Bmal1-KO animals (table 1). Figure 1*d* shows the regression curves delved by Kronos, fitted to the gene expression data along time, for both groups and the examined clock genes. In WT animals, the *Cry2* gene exhibited significant oscillations (*p* < 0.05), indicating a pronounced cyclic pattern within the dataset. Conversely, in Bmal1-KO animals, the oscillations of the *Cry2* gene did not reach statistical significance (*p* = 0.082), suggesting a lack of observed rhythmicity. Despite the rhythmicity of *Rev-erba* gene for both genotypes, with *p*-values of 0.0008 (WT) and 0.018 (Bmal1-KO), the amplitude of this gene in the Bmal1-KO is close to 0, indicating low expression levels. However, pairwise analysis revealed differential rhythmicity between the curves (*p* < 0.01), implying distinct oscillatory patterns despite both groups showing significance in gene oscillation. Neither the *Per2* nor *Clock* genes exhibited significant oscillations in either WT or Bmal1-KO animals, suggesting an absence of discernible cyclic patterns in the expression of these genes within our dataset.

**Table 1.** Kronos circadian rhythmicity analysis data of clock genes.

gene	group	*p*‐value	r.sq	avg	acrophase	amplitude	pairwise *p*‐value
*Per2*	WT	0.15105	0.18042	0.92034	6 82 234	0.30586	1
	Bmal1-KO	0.52649	0.09398	1 23 513	2 03 947	0.13047	
*Cry2*	WT	0.01248	0.38556	0.78217	3 13 735	0.15708	1
	Bmal1-KO	0.08287	0.29937	1 04 455	2 55 370	0.15259	
*Rev-erbɑ*	WT	0.00087	0.54285	0.70612	4 23 198	0.35255	0.002
	Bmal1-KO	0.01798	0.46112	0.11940	4 31 970	0.02056	
*Clock*	WT	0.80448	0.02264	0.86396	2 80 517	0.04304	1
	Bmal1-KO	0.20947	0.21376	1 18 056	3 04 025	0.10491	

### 3.2. Gene expression profile of the Bmal1-KO model

**Figure 2.**
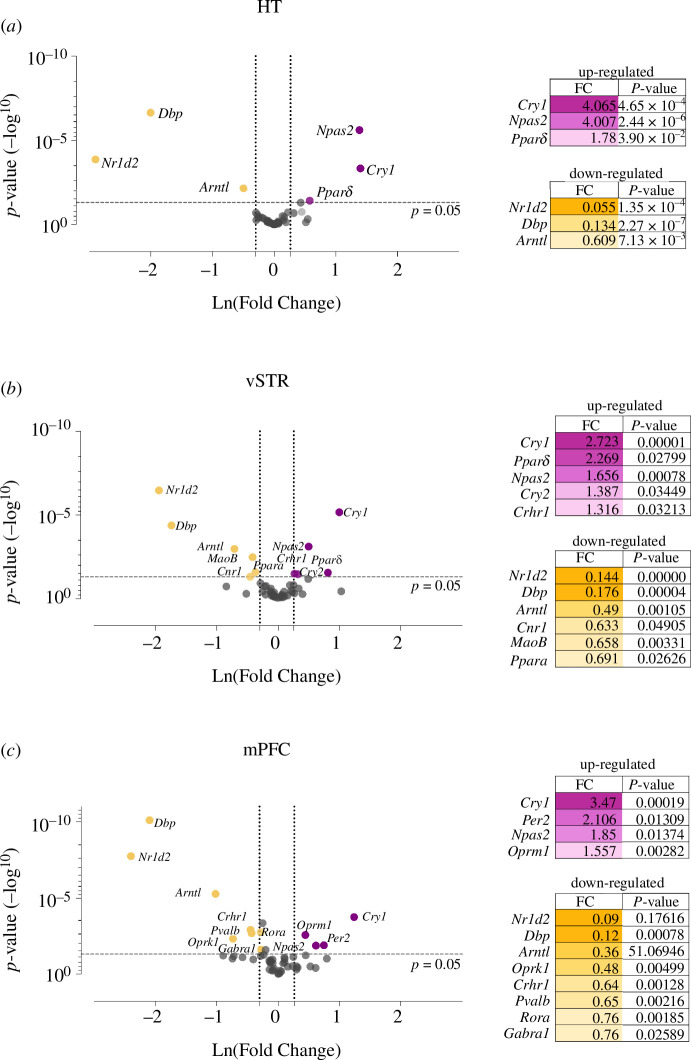
Differentially expressed genes of Bmal1-KO mice compared to WT mice. Comparisons of the expression of 52 genes assessed in OpenArray analysis of mRNA isolated from (*a*) HT, (*b*) vSTR and (*c*) mPFC from WT mice (*n* = 6) and Bmal1-KO mice (*n* = 7). The volcano plot displays the relationship between fold change and significance between the two groups, applying a Student’s *t*‐test. The *y*-axis depicts the negative log^10^ of *p*-values of the *t*-tests (the horizontal slider corresponds to a *p*-value of 0.05) and the *x*-axis is the difference in expression between the two experimental groups as Ln (fold changes) (vertical sliders indicate mRNAs as either up-regulated (right area, purple dots) or down-regulated (left area, yellow dots) from a fold change (FC) of 1.3. The genes that exhibited both an absolute change greater than FC1.3 in either direction with a *p*‐value<0.05 were exposed as the differently expressed genes. HT, hypothalamus; mPFC, medial prefrontal cortex; vSTR, ventral striatum.

We evaluated the gene expression profile of genes related to relevant systems of our interest: clock genes, the reward system and metabolic-related genes in three brain areas such as HT, vSTR and mPFC. The genes that exhibited an absolute change greater than 1.3-fold in either direction with a *p*-value less than 0.05 revealed the different expression for the Bmal1-KO group (figure 2).

Overall, in all nuclei examined, the clock molecular mechanism appears to be dysregulated. Clock genes were generally affected in every region: *Arntl*, *Dbp*, *Nr1d2* down-regulated; *Npas2* and *Cry1* upregulated. Further, in HT we detected the up-regulation of *Ppard* (figure 2*a*). Larger differences in the number of dysregulated genes were observed in the other two regions. Regarding the vSTR (figure 2*b*), the clock gene *Cry2*, was also upregulated. *Ppard* and *Crhr1* also showed significantly higher expression levels in the Bmal1-KO*,* while *Cnr1, Maob* and *Ppara* were down-regulated. In the mPFC (figure 2*c*), we found dysregulation in other genes implicated in circadian rhythms machinery, *Rora* was downregulated and *Per2* was up-regulated. However, genes related to other systems were also disrupted. *Oprm1* was up-regulated, whereas *Oprk1*, *Crhr1*, *Pvalb* and *Gabra1* showed a decreased expression. See electronic the supplementary material, table S4, for the complete list of genes and their statistical analysis.

### 3.3. Daily arrhythmicity alters food and water-induced self-administration behaviour

**Figure 3.**
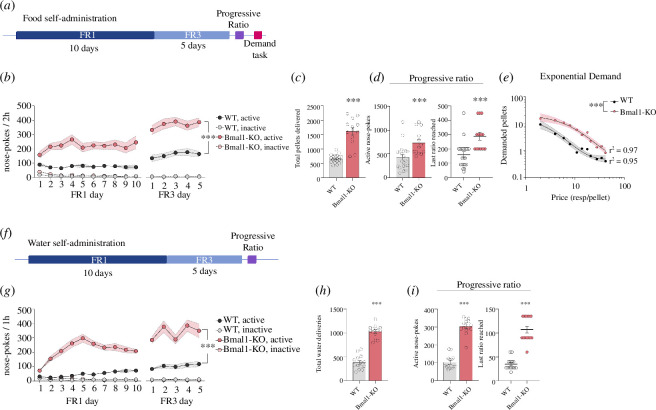
Mice operant training and motivation tests for caloric and non-caloric rewards. (*a*) Schematic outline of the operant training behaviour process for food rewards. (*b*). Active and inactive nose-pokes during FR1 and FR3 for food reward (WT *n* = 22; Bmal1-KO *n* = 14). (*c*) Total pellets delivered through the 15 days of self-administration (SA) procedure. (*d*) Total active nose-pokes and breakpoint (last ratio reached) in the PR test for food. (*e*) Behavioural economic analysis of the demand task and their exponential curve representation of Log of demanded pellets as a function of price. Extra sum-of-squares, ***p < 0.001. (*f*) Schematic outline of the operant training behaviour process for water rewards. (*g*) Active and inactive nose-pokes during FR1 and FR3 for water reward (WT *n* = 14; Bmal1-KO *n* = 14). (*h*) Total water deliveries, obtained through the entire SA procedure. (*i*) Total active nose-pokes and breakpoint (last ratio reached) in the PR test in water SA. Data are represented as mean ± SEM. Two-way repeated measure ANOVA for each FR phase, ***p < 0.001. Student’s *t*‐test, *p < 0.05; **p < 0.01; ***p < 0.001. See the electronic supplementary material, table S2, for a detailed statistical analysis.

We investigated the reward-seeking alterations through the SA paradigm reinforced with a standard pellet of food, in which all mice successfully learned the task regardless of the reinforcement schedules (figure 3*b*). The two-way ANOVA for the active nose-pokes showed significant differences between groups with a much higher seeking behaviour for the food reward in the Bmal1-KO group (*Genotype effect* F(1, 32) = 37.96, *p *< 0.0001). The differences were maintained for a more demanding challenge within the test (FR3) (*Genotype effect* F(1, 32) = 43.53, *p *< 0.0001). The PR test revealed greater levels of motivation evidenced by a significant increase in active nose-pokes (*t*32 = 3.318, *p* < 0.01) and a higher breaking point (*t*32 = 3.734, *p* < 0.001). Also, the Bmal1-KO mice obtained a much larger amount of food pellets overall (*t*20 = 2.872, *p* < 0.001).

Figure 3*e* illustrates the behavioural economics analysis of the demanding task. Economic demand analysis facilitates understanding the role of effort in food procurement and the relationship between physiological and neural mechanisms [35]. The extra sum-of-squares F-test results showed that WT and Bmal1-KO mice exhibited different performance patterns during the task, best represented by two separate demand curves based on the Q0 and α parameters (F(2, 17) = 84, *p* < 0.0001). Both groups exhibited a comparable preferred level of demanded pellets at a minimum price or Q0 (WT: 21,73, 95% CI [7.4, 36]; Bmal1-KO: 22,93, 95% CI [13, 33]). However, Bmal1-KO mice displayed considerably less behavioural elasticity or α (WT: 0.0059, 95% CI [0.0033, 0.0084]; Bmal1-KO: 0.0018, 95% CI [0.0013, 0.0023]). The Pmax value for each data set was higher for Bmal1-KO mice (WT: 2.75; Bmal1-KO: 8,62), suggesting an increased motivation for the reward.

Following the same trend as in food SA, WT and Bmal1-KO mice exposed to water SA exhibited higher seeking behaviour for water independently of the demanding conditions (*Genotype effect* FR1: F(1, 52) = 150.6, *p* < 0.0001; FR3:F(1, 52) = 61.43, *p *< 0.0001). Bmal1-KO mice also obtained a greater amount of water deliveries throughout the operant conditioning protocol (*t*26 = 12.36, *p* < 0.001; figure 3*h*). Eventually, the Bmal1-KO animals presented an elevated number of active responses (*t*26 = 12.95, *p *< 0.001) together with a significantly higher breakpoint in comparison with WT in the PR test (*t*26 = 9.298, *p* < 0.0001; figure 3*i*).

Male and female mice were analysed separately for both paradigms (electronic supplementary material, figure S3). Sex effect was observed in food SA independently of the genotype. However, no interaction between sex and genotype was found for either FR schedules or the PR.

### 3.4. Dopaminergic system is altered in regions associated to reward control in Bmal1-KO mice

We considered further examining the expression of several genes related to feeding behaviour in the HT and reward, particularly to dopaminergic system within the vSTR.

**Figure 4.**
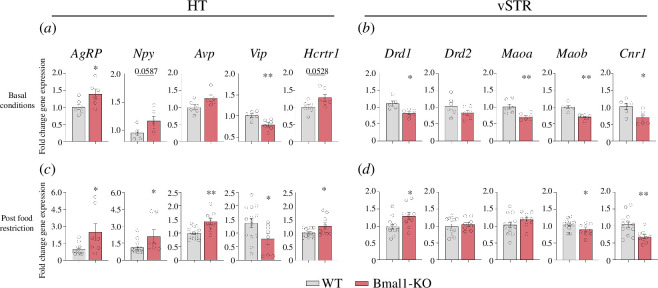
Bmal1-KO mice show altered expression of genes related to food intake control and the reward system in the HT and vSTR, respectively. Gene expression analysis assessed by qPCR in basal conditions (WT *n* = 6; Bmal1-KO *n* = 6) in (*a*) HT and (*b*) vSTR; and after caloric restriction period (WT *n* = 15; Bmal1-KO *n* = 9) in (*c*) HT and (*d*) vSTR. Data are expressed as mean  ±  SEM. Student’s *t*‐test (*p < 0.05; **p < 0.01; ***p < 0.001). HT, hypothalamus; vSTR, ventral striatum.

Notably, we observed nearly identical alterations in gene expression within the HT in both at baseline and after exposure to food restriction period (figure 4*a,c*). Specifically, there was a higher expression in the Bmal1-KO compared to WT in *AgRP* in both basals (*t*10 = 2.277, *p *< 0.05) and post-food restriction (*t*20 = 2.872, *p* < 0.01) conditions. Additionally, there was a trend towards increased expression of *Npy* under basal conditions (*t*9 = 2.164, *p* = 0.0587), with an up-regulation observed after caloric restriction period (*t*20 = 2.589, *p* < 0.05). Further, *Avp* was significantly increased in the Bmal1-KO after the food SA (*t*21 = 3.790, *p* < 0.01) and showed a tendency towards this direction under basal conditions (*t*9 = 2.07, *p* = 0.068). Conversely, *Vip* displayed decreased expression in the Bmal1-KO group under both conditions (basal: *t*10 = 3.345, *p* < 0.01; post-food restriction: *t*20 = 2.259, *p* < 0.05). Lastly, the gene for the hypocretin receptor 1 (*Hcrt1*) showed increased expression in Bmal1-KO animals compared to WT after food restriction (*t*21 = 2.406, *p* < 0.05) and displayed a similar trend under basal conditions (*t*10 = 2.196, *p* = 0.0528).

With respect to the vSTR (figure 4*b,d*), we observed notable differences in expression between the different conditions, depending on the particular gene under study. In both conditions, *Drd2*, *Maob* and *Cnr1* exhibited the same trend, while Drd1 and MAOA did not. Caloric restriction seemed to up-regulate Drd1 expression in the Bmal1-KO group (*t*19 = 2.383, p < 0.05), while it was absent in the ad libitum condition (*t*9 = 3.436, *p* < 0.01). Alternatively, no differences were found in the expression of dopamine receptor 2 (Ddr2) in both conditions (basal: *t10* = 1.489, ns; post-food restriction: *t*18 = 0.6063, ns). Furthermore, MAOA and MAOB were down-regulated in Bmal1-KO mice in basal conditions (*t*10 = 3.301, *p* < 0.01; *t*10 = 5.509, *p* < 0.001). This down-regulation was only mirrored in the food restriction condition for Maob (*t*20 = 2.109, *p* < 0.05). Finally, Cnr1 displayed a downregulation in the Bmal1-KO mice compared to WT in both conditions (basal: *t*10 = 2.755, *p* < 0.05; post-food restriction: *t*20 = 3.076, *p* < 0.01).

## 4. Discusion

In this study, we evaluated the absence of the *Bmal1* gene in mice as a proper model of daily arrhythmia and how this internal dysregulation manifests in terms of activity and motivation for natural rewards. We assessed it through two different innovative techniques to analyse internal rhythmicity: VAE analysis for locomotor activity and the computational tool Kronos to further analyse activity data while assessing time-course gene expression oscillations. This arrhythmia not only affects locomotion but also influences the motivation and reward behaviour of the animals. We further questioned whether this regulation is metabolic in nature or solely involves the reward system.

Our results offer additional evidence that the constitutive Bmal1-KO model used in this study displays disrupted and less predictable activity patterns for several behaviours, including locomotion and motivated behaviours. We used the concept of variation of entropy to describe the degree of organization or complexity in the organism’s locomotor activity pattern. When the organism possesses non-disrupted circadian rhythms (WT animals), the locomotor activity pattern displays a consistent and predictable structure. Therefore, peaks and troughs of the wave occur at approximately the same times every day, and the pattern will repeat in a regular and organized manner [36]. Alternatively, disruption in the organism’s locomotor activity pattern caused by changes in the environment or a disruption in its internal clock, may result in a more random or shifted pattern. Peaks and troughs of the wave occur at different times or in a less predictable manner, resulting in a higher degree of perturbation of the organism’s homeostasis. Moreover, we also observed reduced locomotor activity in Bmal1-KO animals due to the well-described muscle weakness they display [37–39]. In the same line, Smith *et al*. [40] showed impaired locomotor activity and feeding patterns in the same Bmal1-KO model, and [7] similarly demonstrated how another mouse model lacking BMAL1 struggled to adjust to light schedules and was completely arrhythmic under free-running conditions. Similarly, we assessed faint adjustments of the Bmal1-KO to the light-dark schedule, however the arrhythmicity patterns in their spontaneous locomotor activity remain predominant, and the difference in accumulated entropy could not be dismissed. The VAE mathematical method employed here allowed the quantification of the degree of perturbation occurring in these genetically modified mice, with VAE(M) = 0.41 compared to the WT.

Traditional methods for evaluating circadian rhythmicity, such as JTK_CYCLE and Cosinor [41,42] Hughes *et al*., 2010, while proficient, may be more demanding in terms of coding or missing the assessment of differential rhythmicity between groups. In contrast, VAE analysis, in combination with Kronos software analysis offers considerable advantages. They not only allowed the evaluation of differences in acrophase and amplitude like other analytical tools, but this combined methodology also assessed arrhythmicity patterns in terms of quantitative differences. Specifically, VAE analysis evaluates the complexity of the wave pattern describing locomotor activity data series in terms of entropy divergence. This approach allows for a comprehensive comparison of rhythmicity between experimental groups. Given that VAE analysis is a comparative methodology, a positive resultant value indicates that Bmal1-KO mice exhibit more complex circadian dynamics, suggesting a richer informational content in their time series data consistent with their visually desynchronized pattern.

Complexity dynamics related to circadian rhythms are not well established and further analyses would be necessary to evaluate these matters. Eventually, in scenarios where an organism’s circadian rhythms may be disrupted by environmental, genetic or pharmacological factors, this VAE analysis can be used to quantify the extent of the disturbance and compare the impact of different situations on circadian rhythms.

Furthermore, chronobiology research has employed the fluctuation in the expression of clock genes during the day to ascertain the impact of genetic or environmental alterations on the circadian rhythms of animals at a molecular level [43,44]. Concretely, the study of the HT is of particular importance. It contains the SCN, a nucleus that synchronizes the internal circadian timing system with the environment and regulates most circadian rhythms in the organism [4]. Our investigation using Kronos [26] unveiled distinctive gene-specific rhythmicity patterns in the HT within the studied animal groups. Within the HT, the *Cry2* gene showed significant oscillations exclusively in WT animals, whereas *Rev-erba* demonstrated significant oscillations in both WT and Bmal1-KO groups, albeit with significantly different rhythmic patterns probably due to the almost absence of expression of this gene in the Bmal1-KO. Conversely, *Per2* and *Clock* did not exhibit significant oscillatory behaviour in either WT or Bmal1-KO animals. Since HT comprises different nuclei, the possible asynchrony between the rhythmic activity of different cellular groups could interfere with the oscillatory behaviour of *Per2* and *Clock*. However, these findings highlight the differential gene-specific rhythmicity present in WT and Bmal1-KO animals, shedding light onto the distinct oscillatory patterns of key genes and their potential implications in biological processes.

Consistently with the previous data, OpenArray analysis reported a dysregulation in the expression of several clock genes in the three areas of study: HT, mPFC and vSTR. These three areas were selected for their roles in the control of circadian rhythms and metabolic regulation, one of the key nuclei for reward modulation, and action control, respectively. Additionally, these latter two areas receive indirect projections from the SCN via the VTA and lateral habenula [45], forming an intricate circuitry for regulating motivated behaviour in a circadian-dependent manner.

Besides the impairment of the clock genes within the HT, as circadian machinery is directly or indirectly affected by *Bmal1*, the vSTR and mPFC also experienced up and down-regulation of several clock genes, which entails a dysregulation in the molecular circadian machinery. Particularly noteworthy is the general downregulation of the *Nr1d1* gene encoding REV-ERB, as expected since *Bmal1* directly regulates its expression [46], possibly contributing to daily arrhythmicity and behavioural changes. All the alterations derived from the disturbances in this machinery may promote different behavioural alterations and pathological conditions as already reported in other studies [47]. For instance, resembling our results, Porcu *et al*. [47] found high expression of both *Cry1* and *Cry2* in NAc in mice that showed mood alterations. Conversely, the dysregulation in the expression of the other studied genes may be a direct or indirect consequence of the disruption of the circadian molecular clocks. Specifically, overexpression of *Crhr1* may be a compensatory effect of the lower cortisol levels that are intrinsic in the Bmal1-KO line [48].

We intriguingly observed a sustained rise in motivation and seeking behaviour in animals lacking BMAL1 in the SA paradigm. What is especially noteworthy is that we observed this behaviour despite the reduced activity of Bmal1-KO animals. This suggest that the observed differences in motivation may not be attributable to disruptions in the decreased locomotor activity *per se. Bmal1* has previously been implicated in appetitive learning paradigms [49], where its specific deletion in NAc astrocytes increased motivation for food, conversely to the non-altered behaviour in sucrose consumption observed in striatal Bmal1-KO [25]. Our results regarding no sex differences in motivation for natural rewards align with previous studies where no outstanding sex differences were observed. Therefore, considering these collective findings and the absence of previously reported sex differences [50], we opted to collapse both males and females together throughout our studies.

Interestingly, the observed heightened seeking behaviour in Bmal1-KO was consistent regardless of the caloric content of the reward. This led us to further explore the possibility that the reward system is strongly affected by rhythmicity impairments. Consequently, we investigated the dopaminergic system, one of the main systems implicated in reward regulation and influenced by circadian control [14]. We found that dopaminergic signalling might be disturbed in the Bmal1-KO, as evidenced by a decrease in the expression of *Maob*, a DA-degrading enzyme. This, coupled with the high-seeking behaviour displayed by Bmal1-KO mice in food SA procedures, is consistent with previous studies in which inhibition of the enzyme increased the effort required to obtain food rewards [51]. Additionally, we observed alterations in the CB1 receptor gene, an important regulator of feeding and reward [17,52,53]. The interplay of these systems could be contributing to the high motivation observed in these animals. Moreover, we reported dysregulation in *Gabra* and *Pvalb* genes, indicating a possible impairment of the GABAergic system within the mPFC. The dysregulation of this region can impact the ability to control impulses and regulate memory and decision-making [54]. Hence, it is possible that Bmal1-KO mice struggle with reduced self-control, which could result in an altered pursuit of primary rewards beyond basic survival needs, and therefore leading to compulsive-like behaviours. Supporting this hypothesis, our behavioural economic analysis revealed a low demand elasticity in the Bmal1-KO animals, suggesting an impaired ability to adapt to changes in the price of reward and hinting at a potential link between dysregulation of the molecular clock and a compromised behavioural adjustment in response to changing reward conditions. Furthermore, in the mPFC, the opioid system is disbalanced in the absence of BMAL1, possibly influencing the increase in motivation for natural rewards. Notably, we found an upregulation of *Oprm1*, a mu receptor that enhances reward, and downregulation of the *Oprk1* gene, a kappa receptor that promotes aversive behaviour [55] in mPFC.

Even though our research indicates that irregularities in circadian rhythmicity impact gene expression and, in turn, reward processing, it is crucial to recognize that different arrhythmicity models may yield different results. Contrary to our observations, some arrhythmicity models exhibit a decrease in motivation for food reward [56]. Similarly, in previous studies, we reported a decrease in cocaine seeking of Bmal1-KO under an SA paradigm [8]. However, the regulation of drug of abuse and natural rewards could be different and influenced by diverse environmental cues and internal conditions that could explain these apparent discrepancies. For instance, studies on constitutive Bmal1-KO mice [50] have revealed diverse phenotypic alterations, including accelerated ageing, reduced body weight, hypocortisol and hypoinsulinemia [57–60]. In such a case, the metabolic issues may be impacting the motivation for cocaine and food in different ways. Further, regarding our results, the loss of BMAL1 leading to altered glucose [61] and ghrelin metabolism [62] can incentivize food-seeking by modulating the significance of food as a mechanism aimed at maintaining metabolic homeostasis. Moreover, Bmal1-KO mice may be more vulnerable to stress conditions [24], which could lead to higher perception of the reinforcing value of food and a decrease in their impulsive control. However, despite the metabolic impairments of the Bmal1-KO model, the persistent increase in motivation beyond the caloric component of the reward, leads us to hypothesize that BMAL1 may be involved in influencing the reward system. Crucially, studies such as the one conducted by Kolbe and colleagues [2] demonstrate that normalizing circadian rhythms can effectively normalize body weight and glucose metabolism, and could modulate the rewarding significance of food. Most of this emphasizes the importance of the lack of circadian rhythmicity as a key factor in the modulation of behaviour.

Despite the potential impact of metabolic abnormalities resulting from disrupted circadian rhythms in mice, we contend that the primary driver behind the observed behaviours lies in the altered reward system. This assertion gains support from our consistent findings, even when utilizing a non-caloric reinforcement.

To explore whether arrhythmicity-induced metabolic changes contribute to the altered behaviour in Bmal1-KO mice, we examined the expression of *AgRP* and *Npy*. Notably, their increased expression in Bmal1-KO, nearly independent of caloric restriction conditions, aligns with the activation of AgRP neurons. These neurons, co-expressing the orexigenic peptides AgRP and NPY, play an important role in feeding behaviour and project to mesolimbic regions, regulating both reward-related behaviours and eating [63]. Our data also revealed an impact on the orexinergic (ORX) system, with increased *Hprtr1* expression in the HT, consistent with previous findings while indicating clock gene regulation of the ORX system [64]. Moreover, activation of D1 neurons mediates rewarding consumption and seeking, suggesting a pivotal role in the reinforcement process [65]. Interestingly, the overexpression of D1 receptors within the vSTR after food SA, rather than in basal conditions, may be involved in excessive seeking behaviour. This overexpression could perpetuate the hyperactivity of the same neuronal group, leading to the perseveration in seeking the reinforcer.

Recognizing the complexity of the interconnected roles of the circadian system, neurotransmitter control, metabolic homeostasis and reward processing, our comprehensive investigation provides insights into these intricate relationships.

In conclusion, our data illustrate that BMAL1 deficiency significantly disrupts daily rhythms and the cellular molecular clock. This arrhythmia may affect gene expression and, consequently, the proper functioning of the reward system. This interplay results in changes in seeking behaviour and motivation for natural rewards. While our findings provide insight into a specific aspect of this intricate relationship, the observed discrepancies emphasize the necessity of considering various models and their contextual factors. The broader implications highlight the importance of exploring both the environmental and genetic origins of circadian disruptions to comprehensively understand their effects on reward processing. This research lays the foundation for further investigations delving into the dynamic interplay between circadian rhythms and the complex mechanisms governing the reward system.

## Data Availability

Further information and requests for data, resources and reagents should be directed to and will be fulfilled by the corresponding author (O.V.) upon request. Supplementary material is available online [66].
